# In Silico Investigation of Electrically Guided Drug Transport Toward Morphology-Specific Brain Tumor Treatment

**DOI:** 10.3390/pharmaceutics18101300

**Published:** 2026-10-14

**Authors:** Sangwoo Lee, Kyeonggu Lee, Sangjun Lee, Jimin Park, Young Bin Choy, Chang-Hwan Im

**Affiliations:** 1 Department of Electronic Engineering, Hanyang University, Seoul 04763, Republic of Korea; swoo0426@hanyang.ac.kr (S.L.); lkg0324@hanyang.ac.kr (K.L.);; 2 School of Biomedical Convergence Engineering, Pusan National University, Yangsan 50612, Republic of Korea; 3 Department of Biomedical Engineering, Seoul National University College of Medicine, Seoul 03080, Republic of Korea; 4 Institute of Medical and Biological Engineering, Medical Research Center, Seoul National University, Seoul 03080, Republic of Korea; 5 Innovative Medical Technology Research Institute, Seoul National University Hospital, Seoul 03080, Republic of Korea; 6 Department of Biomedical Engineering, Hanyang University, Seoul 04763, Republic of Korea

**Keywords:** brain tumor, glioblastoma, iontophoresis, drug delivery, Nernst–Planck equation, finite element method

## Abstract

**Background/Objectives:** Iontophoresis employs an applied electric field to induce electromigration, enabling the active and directional transport of drug molecules. This study presents the first computational investigation of iontophoretic drug delivery targeting human brain tumors. The objective was to evaluate the feasibility of this approach and to determine how electrode configurations influence drug transport across different tumor morphologies. **Methods:** A realistic finite element head model was constructed from T1-weighted magnetic resonance imaging data. Two representative glioblastoma morphologies were considered: a spherical tumor in the left hemisphere and a butterfly-shaped tumor extending across the corpus callosum. Six electrode configurations spanning three categories (scalp electrodes, combined catheter and scalp electrodes, and catheter electrodes) were systematically evaluated. The electric field distribution was computed by solving the quasi-static Laplace equation, and the spatiotemporal distribution of gadobutrol was obtained using the Nernst–Planck equation. Drug delivery performance was quantified in terms of tumor coverage and targeting efficiency. **Results:** Under the simulated conditions, iontophoresis markedly enhanced drug delivery compared with passive diffusion, increasing tumor volume coverage from 13% to 84% in the spherical tumor model. Scalp electrode configurations resulted in the lowest tumor coverage in both tumor models. The combined catheter–scalp configuration generated radial drug transport from the catheter and achieved the highest coverage in the spherical tumor. In the butterfly-shaped tumor model, a three-catheter configuration enabled bilateral delivery along the tumor axis, achieving a tumor coverage of 49% and a targeting efficiency of 82%. The maximum electric field magnitude across all configurations was 49.26 V/m, remaining below reported axonal activation thresholds for deep brain stimulation. **Conclusions:** These findings support the feasibility of iontophoretic drug delivery to human brain tumors and demonstrate that the effective electrode configuration depends on tumor morphology. This study provides a computational foundation for morphology-specific planning of iontophoretic drug delivery to brain tumors.

## 1. Introduction

Glioblastoma multiforme (GBM) is the most frequent and malignant primary brain tumor, characterized by high mortality and resistance to conventional treatments [1,2]. The current standard of care typically involves surgical resection followed by adjuvant chemoradiotherapy to improve survival rates [3]. In this approach, chemotherapy plays a pivotal role in eradicating infiltrative tumor cells that remain in the surrounding brain parenchyma after surgery, which are the primary cause of recurrence [4]. Moreover, for patients with recurrent GBM or tumors located in eloquent areas where surgical intervention is not feasible, chemotherapy often serves as the primary treatment option [5,6]. However, conventional systemic chemotherapy, typically administered orally or via intravenous injection, is significantly limited by the blood–brain barrier (BBB), which restricts the delivery of therapeutic agents to both the tumor core and the peritumoral regions [7]. Consequently, conventional delivery methods often fail to achieve sufficient drug concentrations at the target site, leading to inevitable recurrence and poor outcomes [8].

To resolve this, convection-enhanced delivery (CED) has been widely adopted in clinical settings as an alternative delivery strategy, where therapeutic agents are directly infused through a catheter inserted into the brain under a hydrostatic pressure gradient [9,10]. However, its clinical efficacy is often constrained by inherent physical limitations, including reflux along the catheter track and unpredictable drug distribution governed primarily by passive convection and diffusion within heterogeneous tissue [9,11]. Crucially, CED provides limited control over drug transport pathways within the tissue [12], which can result in incomplete target coverage or unintended exposure of surrounding healthy tissue. This limitation may be particularly relevant in cases involving elongated or bilaterally extending tumors, where achieving adequate coverage requires drug transport along the tumor geometry.

To address these limitations, our previous study proposed iontophoresis as a promising modality for localized drug delivery within the brain [13]. By applying an electric field via electrodes inserted into the brain, this technique enabled the active and directional transport of the model compound, gadobutrol, through electromigration, as demonstrated in both ex vivo and in vivo rat models. These investigations demonstrated that the imposition of an electric field induces directional transport of gadobutrol from the cathode toward the anode within the rat brain, exhibiting a distinct migration pattern compared to passive diffusion. Furthermore, the extent of drug migration was found to be positively correlated with the duration of current injection, confirming that the transport kinetics and spatial distribution of the drug can be effectively modulated by controlling electrical parameters. Crucially, these empirical findings were successfully corroborated by finite element method (FEM)-based numerical simulations, which accurately reproduced the drug distribution patterns observed in the rat brain. The high concordance between the experimental and computational results indicates that in silico modeling can serve as a robust predictive tool for estimating drug migration paths and diffusion profiles under varying electrical current conditions. This validation provides a strong rationale for employing computational simulations to optimize electrode configurations and explore feasibility in complex anatomical environments, such as human brain tumors, prior to clinical translation.

In this study, to the best of our knowledge, we present the first computational investigation into the feasibility of iontophoretic drug delivery for human brain tumors. We developed three-dimensional models of glioblastomas within a realistic human head model, focusing on two representative morphologies: a compact unilateral lesion (implemented as a spherical geometry) [14] and a butterfly glioblastoma (bGBM) with bilateral extension through the corpus callosum [15]. The bGBM is defined by contrast-enhancing tumor crossing the corpus callosum and involving both hemispheres [16,17]. For each tumor morphology, we systematically simulated the spatiotemporal distribution of drugs under various electrode configurations. Through this comparative analysis, we aimed to determine how electrode placement influences drug transport and to identify effective configurations for each tumor type.

## 2. Materials and Methods

### 2.1. Realistic Head Model

**Figure 1:**
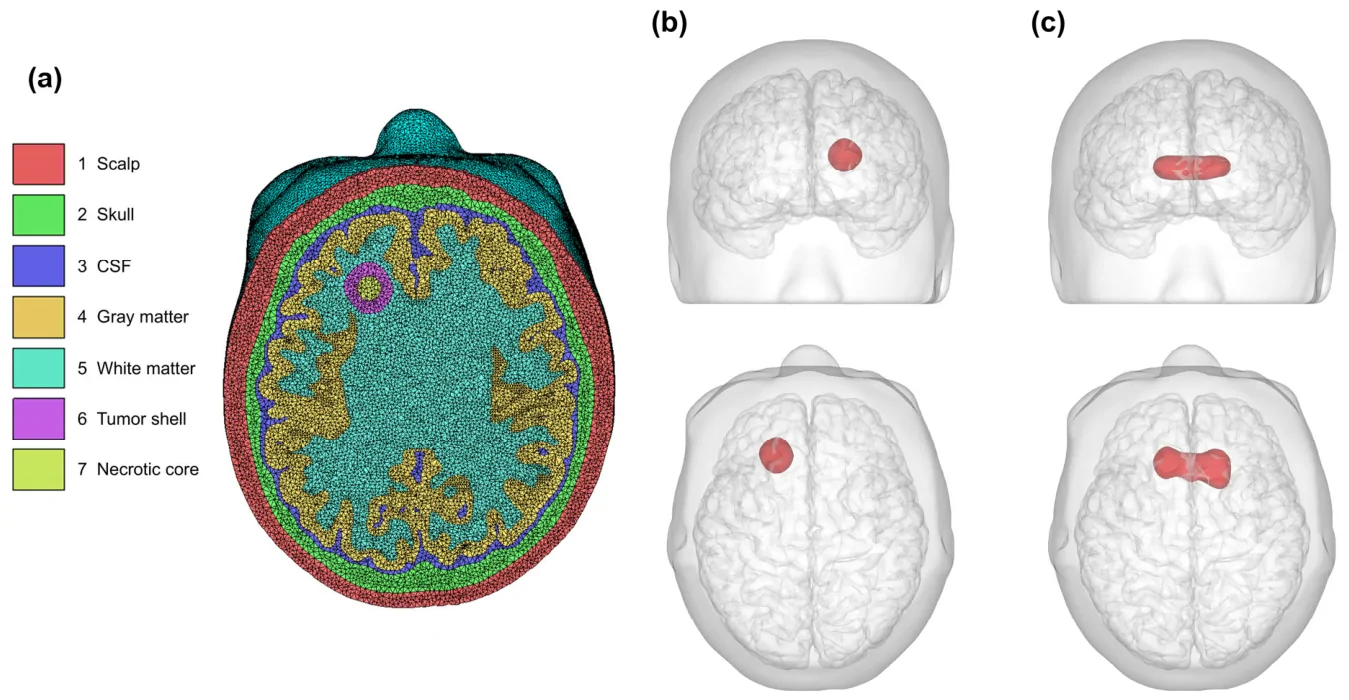
Realistic head model and tumor geometries. (**a**) Structure of the head model comprising seven segmented regions: scalp, skull, CSF, gray matter, white matter, tumor shell, and necrotic core. (**b**) Spherical tumor model shown in coronal (top) and transverse (bottom) views. (**c**) Butterfly-shaped tumor model shown in coronal (top) and transverse (bottom) views.

A realistic finite element head model was constructed from T1-weighted MRI data of a male subject (26 yrs) with no clinical history of psychiatric disorders. The MRI data used in the present study were originally acquired under a research protocol approved by the Institutional Review Board (IRB) Committee of Hanyang University (IRB No. HYI-17-180-5), with written informed consent from the subject. As this study used a single realistic head model for computational feasibility analysis, predefined inclusion and exclusion criteria were not applied. The MRI data were acquired using a 3T MAGNETOM Trio scanner (Siemens, Erlangen, Germany) with a spatial resolution of 1 × 1 × 1 mm^3^. Five tissue types (scalp, skull, cerebrospinal fluid (CSF), gray matter, and white matter) were automatically segmented using SimNIBS v2.0 [18]. Tumor surfaces were then segmented within the white matter using ITK-SNAP v3.8 [19]. Each tumor model comprised two regions: a necrotic core surrounded by a thick shell of active tumor tissue. Two tumor morphologies were modeled based on previously reported GBM morphologies [20,21]: a spherical tumor located in the left hemisphere, and a butterfly-shaped tumor extending through the corpus callosum, as illustrated in Figure 1b and Figure 1c, respectively. The tumor volumes were 4.027 cm^3^ and 8.973 cm^3^ for the spherical and butterfly-shaped models, respectively. The seven segmented surfaces (scalp, skull, CSF, gray matter, white matter, tumor shell, and necrotic core) were merged into a complete head model using an in-house MATLAB 2018b (The MathWorks, Inc., Natick, MA, USA) script. Segmentation errors were corrected, and tetrahedral mesh quality was enhanced by removing isolated nodes and self-intersecting elements [22]. The resulting head model is shown in Figure 1a.

### 2.2. Governing Equations

#### 2.2.1. Electric Field Calculation

The electric field distribution within the head model was computed using an in-house FEM solver developed in Fortran 90 [23]. The solver employs the quasi-static Laplace equation:

(1) **∇** ∙ (*σ ***∇**
*ϕ *) = 0,

where *σ* [S/m] and *ϕ* [V] denote the electrical conductivity and the electric potential, respectively. The electrical conductivity assigned to each segmented region is summarized in Table 1 [24,25]. All tissues were assumed to be homogeneous and isotropic. Dirichlet boundary conditions of 1 V and 0 V were imposed on the exposed surfaces of the anode and cathode electrodes. The computed electric potential distribution was then linearly scaled to yield the desired injection current through the anode surface. For configurations involving more than two electrodes, the electric field was computed separately for each electrode pair, and the resulting fields were scaled and summed based on the superposition principle [26]. The specific injection current and electrode pairing for each configuration are described in Section 2.3.

#### 2.2.2. Drug Transport Simulation

Drug transport simulations were performed under two conditions: passive diffusion without an applied electric field, and iontophoresis with electric fields generated by various electrode configurations. The spatiotemporal distribution of gadobutrol was computed by solving the Nernst–Planck equation [27]:

(2) $$\frac{\partial c}{\partial t}-\alpha \nabla \cdot \left(D\nabla c+\gamma \frac{\mathrm{zFD}}{\mathrm{RT}}c\nabla \phi \right)=0,$$

where *c* [kg/m^3^] represents the concentration of gadobutrol, *D* [m^2^/s] is the diffusion coefficient, *z* is the charge number of the gadobutrol molecule, *F* [C/mol] is the Faraday constant, *R* [J/(molK)] is the universal gas constant, *T* [K] is the absolute temperature, α is the time factor, and *γ* is the charge factor. The convective flow effects were neglected following the existing literature [28,29]. The simulation parameters were primarily based on values employed in a previous simulation study using a rat brain model [13]. For gray matter, white matter, and tumor shell, *D* was set to 2.2 × 10^−10^ m^2^/s based on the averaged apparent diffusion coefficients of gadobutrol in the rat brain tissue. For CSF and necrotic core, *D* was adjusted based on the differences in tortuosity (*λ*) among tissue types, since *D* is inversely proportional to *λ*^2^. Considering the reported tortuosity values for CSF, glioblastoma cellular regions, and necrotic regions [30,31], the relative ratio of *D* for CSF, tumor shell, and necrotic core was approximated as 4:1:2. Accordingly, *D* was set to 8.8 × 10^−10^ m^2^/s for CSF and 4.4 × 10^−10^ m^2^/s for necrotic core. *D* was set to zero for electrodes, scalp, and skull, as the electrodes are solid conductors impermeable to drug molecules, and drug transport to the scalp and skull regions was considered negligible. The diffusion coefficients for all regions are summarized in Table 1. The charge number *z* was set to −1 to represent the anionic form of gadobutrol. Although gadobutrol is predominantly neutral under physiological conditions, approximately 5.7% has been reported to exist in an anionic form [32]. Therefore, *z* = −1 was used as a nominal charge number to represent the direction of electromigration rather than the net charge of the entire gadobutrol population. The empirical correction parameters *α* and *γ* were set to 0.5 and 25, respectively, according to our previous study [13]. These correction parameters were originally calibrated against in vivo experimental data obtained from rat brains; however, due to the practical difficulty of experimentally determining these parameters directly in human brain tissue, the same values were adopted in the present study.

The injection region was defined as a rectangular volume with dimensions of 2 × 3 × 6 mm^3^. A constant gadobutrol concentration of 0.01 kg/m^3^ was maintained in this region throughout the simulation [13]. This boundary condition represents an idealized sustained drug supply from the administration site rather than a finite bolus dose. The location of the injection region was determined based on the electrode configuration. The simulation was performed with a time step of 1 s. The total simulation time was set to 180 min for the spherical tumor model and 300 min for the butterfly-shaped tumor model, reflecting the larger tumor volume and longer transport distance of the latter.

### 2.3. Electrode Configurations

Six electrode configurations were designed, including two for the spherical tumor model and four for the butterfly-shaped tumor model. In the spherical tumor model, passive diffusion without an applied electric field was also simulated as a baseline condition. These configurations were categorized into three types: scalp electrodes, a combination of catheter and scalp electrodes, and catheter electrodes. Each scalp electrode was modeled as a disc with a diameter of 10 mm and a height of 2 mm [33]. Each catheter electrode was modeled as a cuboid with dimensions of 0.2 × 2 × 6 mm^3^ [13]. A current was injected through the two largest faces (2 × 6 mm^2^) of the catheter electrode.

#### 2.3.1. Scalp Electrode Configurations

**Figure 2:**
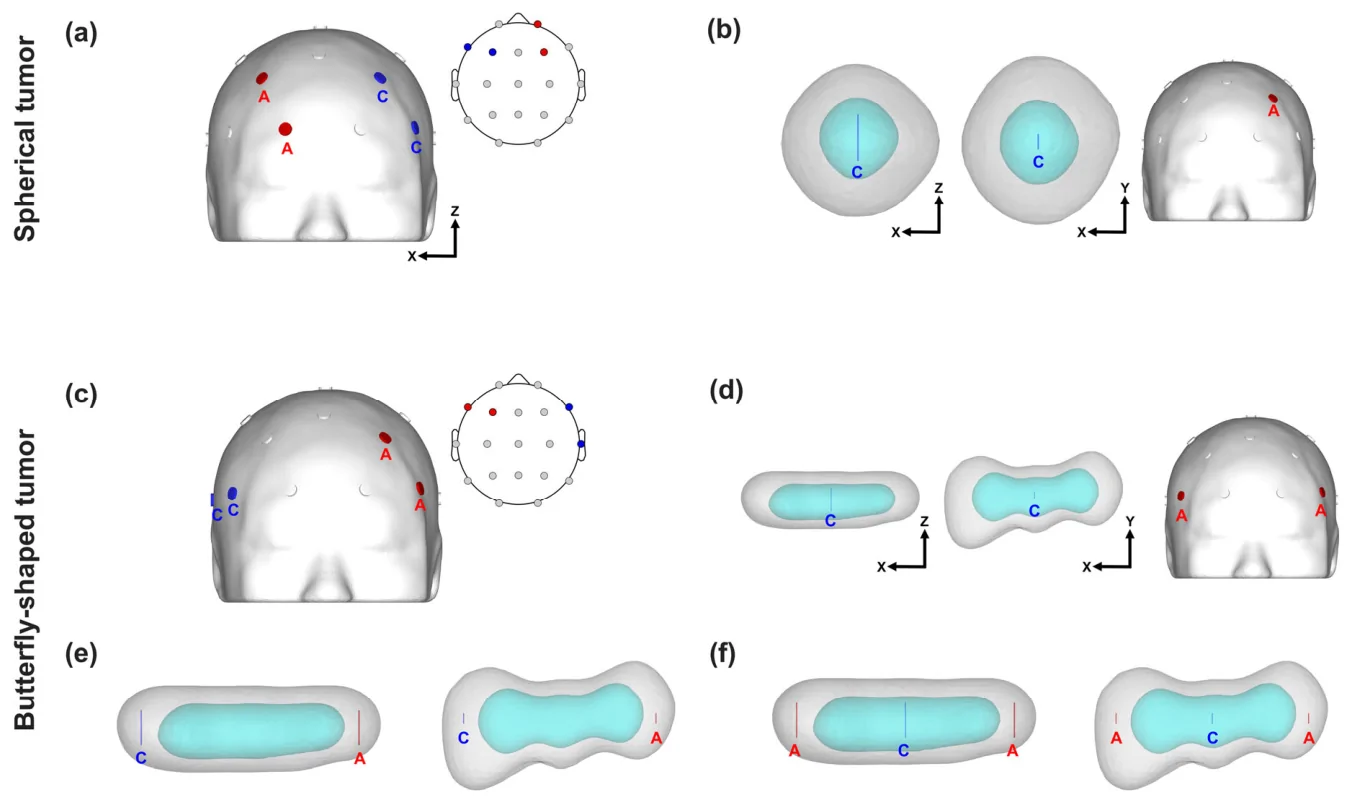
Electrode configurations for the spherical and butterfly-shaped tumor models. Red and blue markers indicate anodes (A) and cathodes (C), respectively. The total injection currents were 2 mA for scalp electrode configurations and 50 μA for configurations involving catheter electrodes. (**a**) Optimized scalp electrode configuration for the spherical tumor model with the 10–20 system electrode layout. (**b**) One catheter electrode at the tumor center with a scalp anode at F3. (**c**) Optimized scalp electrode configuration for the butterfly-shaped tumor model with the 10–20 system electrode layout. (**d**) One catheter electrode at the tumor center with scalp anodes at F7 and F8. (**e**) Two-catheter configuration with a cathode at the right end and an anode at the left end of the tumor. (**f**) Three-catheter configuration with a central cathode and two lateral anodes.

For scalp electrode configurations, 19 electrodes were placed on the scalp according to the international 10–20 electroencephalography electrode system. The optimal injection current pattern was determined by solving a convex optimization problem formulated for each tumor morphology. In the spherical tumor model, the drug injection region was positioned at the center of the tumor. The objective function was set to maximize the inward-directed electric field component on the necrotic core surface:

(3) $$\max_{I}\int_{\Omega_{\mathrm{NC}}}E(r)\cdot \hat{n}\left(r\right)dr,$$

where **E**(**r**) represents the electric field at location **r**, $\hat{n}\left(r\right)$ is the inward-pointing unit normal vector on the necrotic core surface, and *Ω*_NC_ denotes the necrotic core surface. This formulation was designed to drive the anionic form of gadobutrol radially outward from the tumor center toward the surrounding tumor shell. For the butterfly-shaped tumor model, the drug injection region was positioned near the right end of the tumor. The objective function was set to maximize the rightward electric field component in the drug injection region:

(4) $$\max_{I}\int_{\Omega_{\mathrm{inj}}}E(r)\cdot \hat{d}dr,$$

where $\hat{d}$ is the unit vector pointing in the rightward direction and *Ω*_inj_ denotes the drug injection region. Because the anionic form of gadobutrol migrates opposite to the electric field direction, this formulation drives drug transport toward the contralateral (left) end of the tumor. Three constraints were imposed on the injection current vector **I**:

(5a) ‖**I**‖_1_ ≤ 2*s*_tot_,

(5b) ‖**I**‖_∞_ ≤ *s*_indi_,

(5c) $$\sum_{j=1}^{N}I_{j}=0,$$

where ‖∙‖_1_ and ‖∙‖_∞_ denote the *L*_1_-norm and the infinity norm, respectively. The total injection current amplitude was limited to *s*_tot_ = 2 mA, and the maximum current amplitude at each individual electrode to *s*_indi_ = 1 mA. These values were adopted based on established safety guidelines for transcranial electrical stimulation (tES) [34]. Constraint (5c) ensures that the sum of all electrode currents is zero. The optimization problem was solved using CVX v2.1 implemented in MATLAB [35]. The resulting optimized configurations are illustrated in Figure 2a,c. The spherical tumor model yielded anodes at Fp2 and F4 (1 mA each) and cathodes at F3 and F7 (−1 mA each), while the butterfly-shaped tumor model resulted in anodes at F3 and F7 (1 mA each) and cathodes at F8 and T4 (−1 mA each).

#### 2.3.2. Combined Catheter and Scalp Electrode Configurations

For configurations with both catheter and scalp electrodes, a catheter electrode was inserted at the center of the tumor as a cathode. A current was applied to both faces of the catheter to promote bidirectional drug transport. The drug injection region was positioned adjacent to the catheter electrode. Because the thin catheter electrode generates high electric field intensity near its edges, a substantially lower current was used compared to scalp electrode configurations. The total injection current was set to a nominal value of 50 μA, with reference to the 10 μA current used in our previous rat model study [13]. This value was used as a modeling condition rather than a clinically established current level. In the spherical tumor model, the scalp anode was positioned at F3, the closest electrode to the tumor, to maximize radial drug transport from the tumor center (Figure 2b). A current of −50 μA was applied to the catheter and +50 μA to the scalp anode. In the butterfly-shaped tumor model, two scalp anodes were placed at F7 and F8 to direct drug transport bilaterally along the longitudinal axis of the tumor (Figure 2d). A current of −50 μA was applied to the catheter, with +25 μA at each scalp anode.

#### 2.3.3. Catheter Electrode Configurations

Catheter electrode configurations were designed for the butterfly-shaped tumor model. The same total injection current of 50 μA was applied. In the two-catheter configuration, a cathode was placed at the right end and an anode at the left end of the tumor (Figure 2e). A current was applied only to the medial face of each electrode to promote directional drug transport from the cathode toward the anode. The drug injection region was positioned near the right end of the tumor, slightly inward from the cathode. A current of −50 μA was applied to the cathode and +50 μA to the anode. In the three-catheter configuration, a central cathode and two lateral anodes were placed along the longitudinal axis of the tumor (Figure 2f). A current was applied to both faces of the central cathode and to the medial face of each lateral anode. The drug injection region was positioned at the center of the tumor adjacent to the central cathode. A current of −50 μA was applied to the central cathode, with +25 μA at each lateral anode.

### 2.4. Evaluation Metrics

Two metrics were used to quantitatively evaluate drug delivery efficacy across different electrode configurations: tumor coverage (TC) and targeting efficiency (TE). TC was defined as the fraction of tumor volume in which the drug concentration exceeded a specified threshold relative to the total tumor volume:

(6) $$\mathrm{TC}=\frac{V_{\mathrm{th}}}{V_{\mathrm{tumor}}},$$

where *V*_th_ denotes the volume of the tumor region where the drug concentration exceeds the threshold concentration *c*_th_, and *V*_tumor_ denotes the total tumor volume including both the tumor shell and necrotic core. The concentration threshold was set to 0.001 kg/m^3^, corresponding to 10% of the initial concentration (0.01 kg/m^3^). The 10% threshold was used only as a computational criterion for comparing concentration coverage among configurations and does not represent a pharmacologically effective concentration. TE was defined as the ratio of the drug mass within the tumor to the total drug mass distributed throughout the head model:

(7) $$\mathrm{TE}=\frac{M_{\mathrm{tumor}}}{M_{\mathrm{total}}},$$

where *M*_tumor_ is the total drug mass within the tumor and *M*_total_ is the total drug mass in the entire head model. TC and TE were evaluated at 30 min intervals throughout the simulation, with final values reported at 180 min for the spherical tumor model and 300 min for the butterfly-shaped tumor model. Because each simulation condition yielded a single deterministic outcome in one head model, inferential statistical tests were not performed.

### 2.5. Sensitivity Analysis

Sensitivity analyses were performed to evaluate the robustness of the simulation results to the concentration threshold, transport parameters, and tumor conductivity. The sensitivity of TC to the concentration threshold was evaluated using thresholds of 5%, 10%, and 20% of the initial concentration. To evaluate the effects of the transport parameters, the time factor (*α*) and charge factor (*γ*) were varied independently to 50%, 100%, and 150% of their nominal values (*α* = 0.5 and *γ* = 25). When one factor was varied, the other was held at its nominal value. The effects of tumor conductivity were also evaluated by independently varying the conductivities of the tumor shell and necrotic core. The tumor shell conductivity was varied among 0.15, 0.24, and 0.50 S/m, while the necrotic core conductivity was varied among 0.50, 1.00, and 1.50 S/m, based on the minimum, standard, and maximum values reported in a previous computational study of glioblastoma [36]. When one conductivity was varied, all other conductivity values were held at their nominal values. Electrode locations and applied current distributions were fixed to those used under the nominal conductivity condition. All sensitivity analyses were evaluated at the final simulation times of 180 min for the spherical tumor model and 300 min for the butterfly-shaped tumor model. TC was evaluated for the concentration threshold analysis, while both TC and TE were evaluated for the transport parameter and conductivity analyses.

## 3. Results

### 3.1. Electric Field Distributions

**Figure 3:**
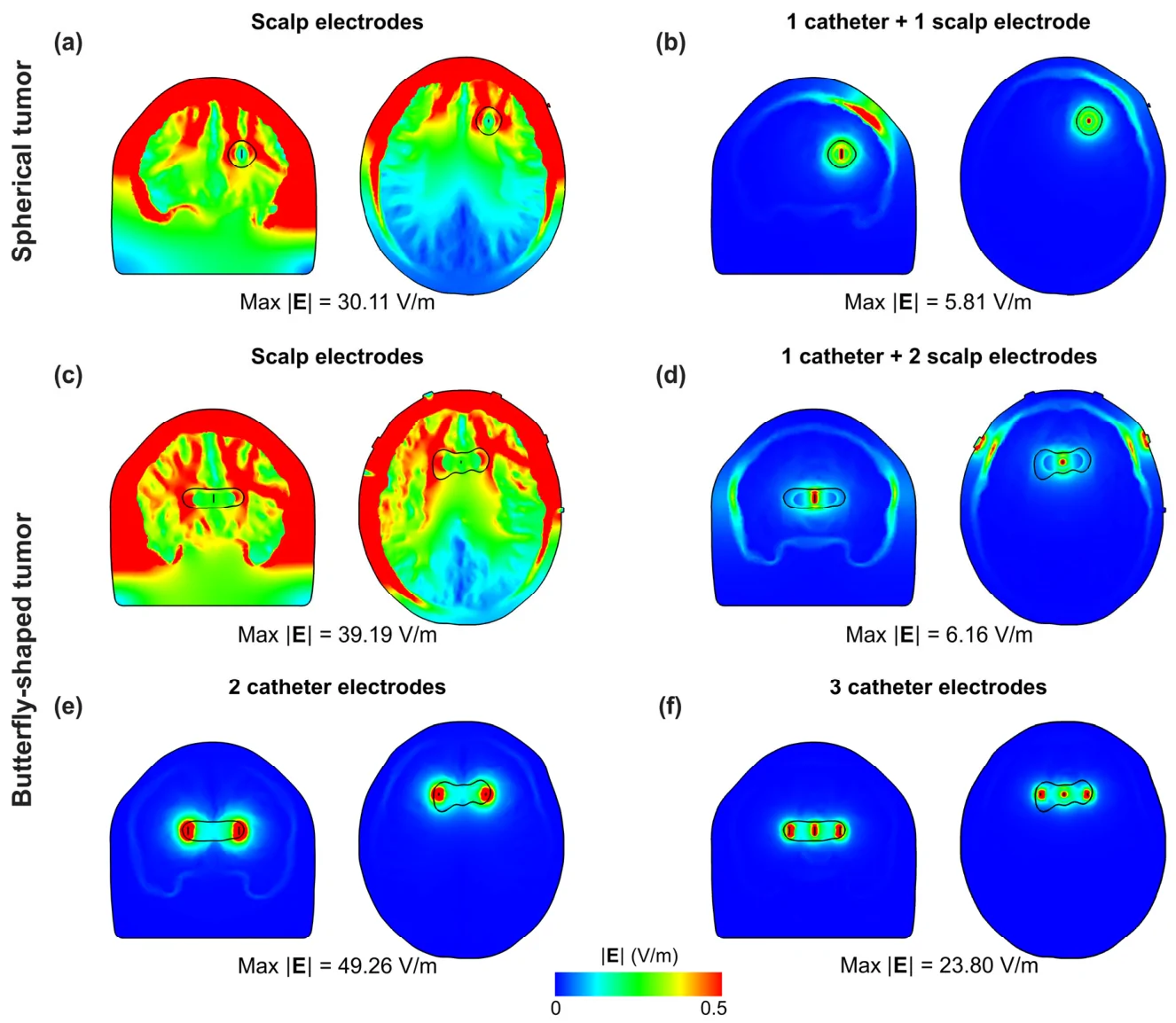
Electric field distributions under the six electrode configurations. Each panel shows coronal (left) and transverse (right) cross-sections, with the tumor boundary outlined in black. The color scale is limited to 0.5 V/m for visualization of the overall distribution. The maximum electric field magnitude across the entire head model is indicated below each panel. (**a**) Scalp electrode configuration for the spherical tumor model. (**b**) Combined catheter and scalp electrode configuration for the spherical tumor model. (**c**) Scalp electrode configuration for the butterfly-shaped tumor model. (**d**) Combined catheter and scalp electrode configuration for the butterfly-shaped tumor model. (**e**) Two-catheter configuration for the butterfly-shaped tumor model. (**f**) Three-catheter configuration for the butterfly-shaped tumor model.

The electric field distributions for all six electrode configurations are presented in Figure 3. The scalp electrode configurations generated broadly distributed electric fields throughout the brain (Figure 3a,c), with the highest intensities observed in scalp and skull regions. The maximum electric field magnitudes were 30.11 V/m for the spherical tumor model and 39.19 V/m for the butterfly-shaped tumor model. Within the tumor region, substantially lower intensities were observed compared to the superficial layers.

The combined catheter and scalp electrode configurations (Figure 3b,d) produced more localized electric fields, primarily beneath the scalp electrode and around the catheter electrode. In both tumor models, the field around the catheter was radially distributed. The maximum electric field magnitudes were 5.81 V/m and 6.16 V/m for the spherical and butterfly-shaped tumor models, the lowest among the three configuration types.

The catheter electrode configurations (Figure 3e,f) produced the most spatially selective electric fields, confined predominantly within the tumor. The two-catheter configuration yielded the highest maximum electric field magnitude of 49.26 V/m among all configurations, while the three-catheter configuration produced 23.80 V/m.

### 3.2. Drug Distribution in the Spherical Tumor Model

**Figure 4:**
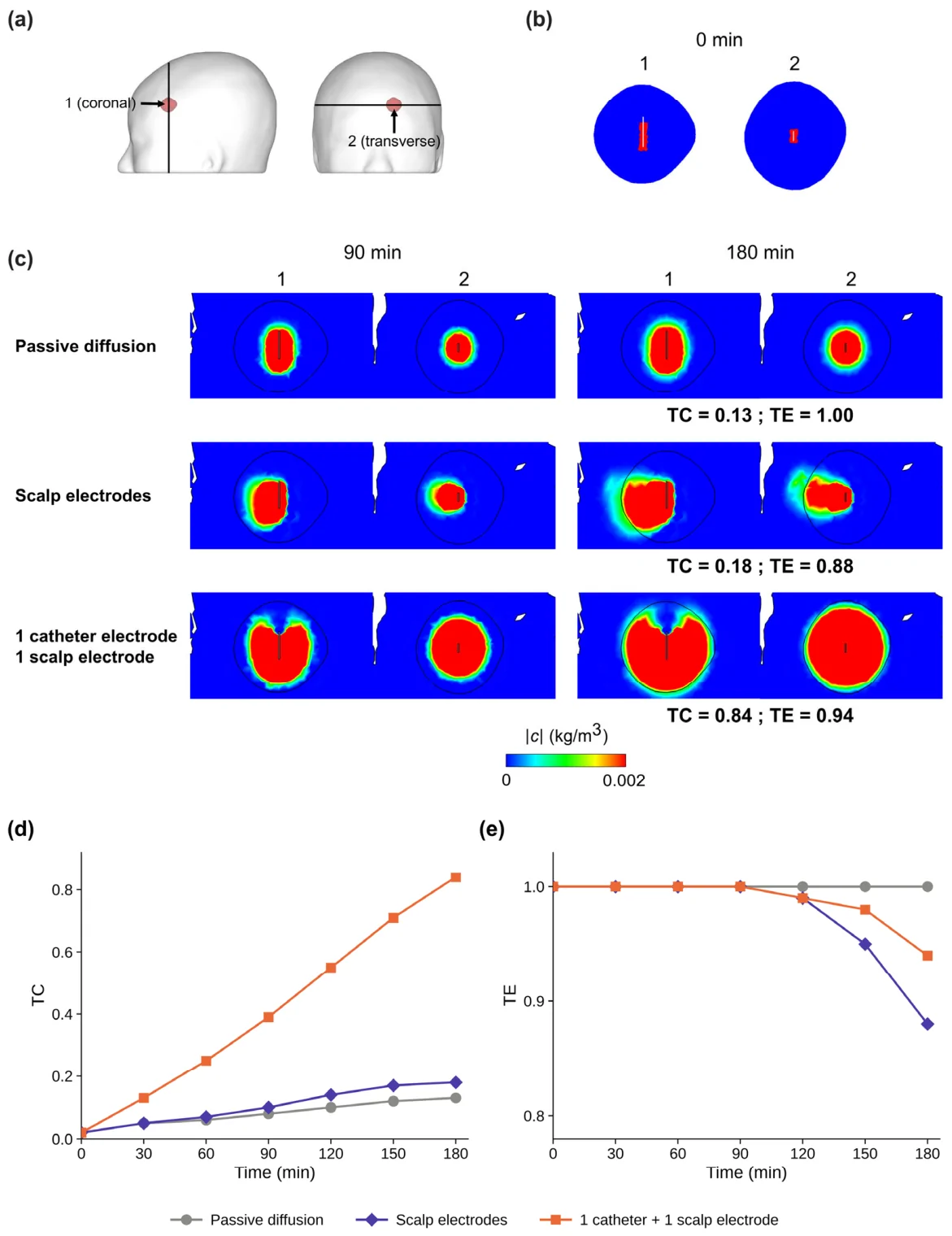
Drug distribution in the spherical tumor model under passive diffusion and the two electrode configurations. (**a**) Cross-section planes used for visualization, with the tumor displayed as a red volume. The arrows indicate the direction of view for each cross-section. 1: coronal, 2: transverse. (**b**) Initial drug distribution at 0 min. The red area indicates the injection region with a concentration of 0.01 kg/m^3^. (**c**) Drug concentration distributions at 90 min and 180 min, shown in enlarged views of the tumor region. The tumor coverage (TC) and targeting efficiency (TE) values at 180 min are indicated below each row. (**d**) Time courses of TC and (**e**) TE, evaluated at 30 min intervals throughout the 180 min simulation period.

Figure 4 illustrates the spatiotemporal drug distributions in the spherical tumor model under passive diffusion and the two electrode configurations. Under passive diffusion, the drug spread uniformly in all directions from the drug injection region. However, the extent of diffusion remained limited relative to the total tumor volume, resulting in a TC of 0.13 and a TE of 1.00. Although the scalp electrode configuration increased TC slightly to 0.18, the drug migrated preferentially in one direction and partially beyond the tumor, reducing TE to 0.88. In the configuration with the catheter cathode at the tumor center and a scalp anode at F3, the drug spread broadly throughout the tumor volume. This configuration yielded the highest TC of 0.84 among the three conditions, with a TE of 0.94, comparable to that of passive diffusion. The time-course analysis showed that the TC advantage of the combined catheter and scalp electrode configuration became progressively larger throughout the simulation (Figure 4d). TC reached 0.39 at 90 min and 0.84 at 180 min, whereas the passive diffusion and scalp electrode conditions remained below 0.20 throughout the simulation. TE remained close to 1.0 during the early period and decreased modestly to 0.94 at 180 min (Figure 4e).

### 3.3. Drug Distribution in the Butterfly-Shaped Tumor Model

**Figure 5:**
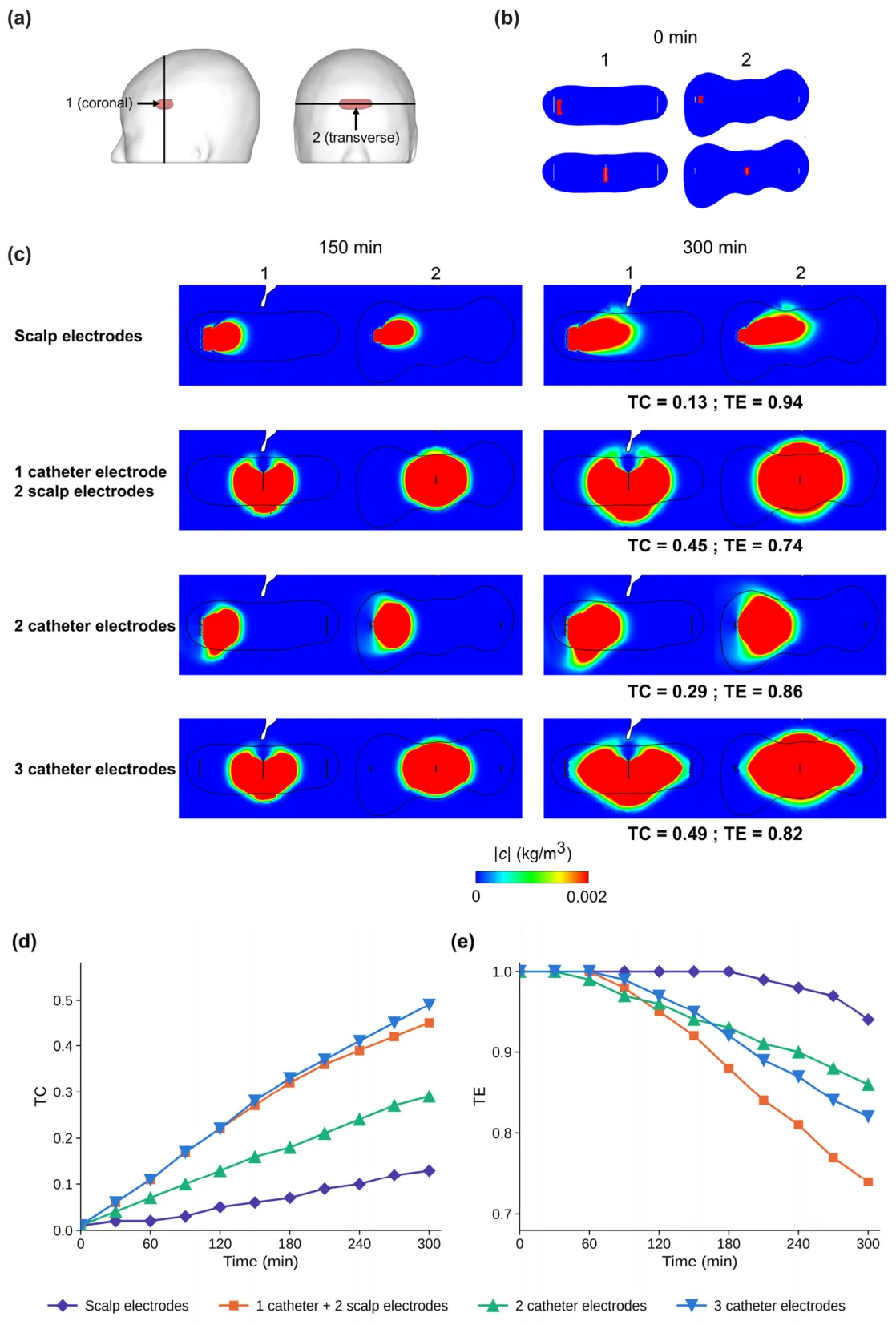
Drug distribution in the butterfly-shaped tumor model under the four electrode configurations. (**a**) Cross-section planes used for visualization, with the tumor displayed as a red volume. The arrows indicate the direction of view for each cross-section. 1: coronal, 2: transverse. (**b**) Initial drug distribution at 0 min. The top and bottom rows correspond to injection regions near the right end and at the center of the tumor, respectively. The red area indicates the injection region with a concentration of 0.01 kg/m^3^. (**c**) Drug concentration distributions at 150 min and 300 min, shown in enlarged views of the tumor region. The first and third rows used the right-end injection region, and the second and fourth rows used the central injection region. The tumor coverage (TC) and targeting efficiency (TE) values at 300 min are indicated below each row. (**d**) Time courses of TC and (**e**) TE, evaluated at 30 min intervals throughout the 300 min simulation period.

Figure 5 presents the spatiotemporal drug distributions in the butterfly-shaped tumor model under the four different electrode configurations. The scalp electrode configuration induced drug transport along the longitudinal axis of the tumor. However, the extent of migration was limited, resulting in a small TC of 0.13 and a TE of 0.94. In the configuration with the catheter cathode at the tumor center and scalp anodes at F7 and F8, TC increased to 0.45. Despite this higher coverage, the drug spread radially from the catheter and extended beyond the tumor boundary perpendicular to the longitudinal axis. This resulted in the lowest TE of 0.74 among all four configurations. The two-catheter configuration produced directional drug transport from the drug injection region toward the anode, with an increased TC of 0.29 and a TE of 0.86. However, the drug did not reach the contralateral end of the tumor at the end of the simulation. The three-catheter configuration achieved the highest TC of 0.49 among all configurations for the butterfly-shaped tumor model. The drug spread bilaterally from the central cathode along the longitudinal axis of the tumor, with a fairly high TE of 0.82. The time-course analysis showed that the three-catheter configuration maintained a slightly higher TC than the combined catheter and scalp electrode configuration throughout most of the simulation (Figure 5d). The three-catheter configuration also maintained higher TE than the combined catheter and scalp electrode configuration over time (Figure 5e).

### 3.4. Sensitivity Analysis Results

Sensitivity analyses showed that variations in the evaluated parameters affected the absolute TC and TE values, while the main configuration-dependent trends were preserved. Varying the concentration threshold from 5% to 20% of the initial concentration changed the absolute TC values but did not alter the configuration with the highest TC in either tumor model (Table S1). Across variations in the time factor (*α*) and charge factor (*γ*), the combined catheter and scalp electrode configuration consistently yielded the highest TC in the spherical tumor model, whereas the three-catheter configuration yielded the highest TC in the butterfly-shaped tumor model (Figure S1 and Table S2). The same configuration-dependent trends were maintained when the conductivities of the tumor shell and necrotic core were varied (Figure S2 and Table S3).

## 4. Discussion

This study presented the first computational investigation of iontophoretic drug delivery targeting human brain tumors. Two representative tumor morphologies were modeled, and six electrode configurations were systematically evaluated using FEM-based numerical simulations. The results showed that iontophoresis substantially enhanced drug delivery efficacy compared to passive diffusion, increasing TC from 0.13 to 0.84 in the spherical tumor model. We also confirmed that drug transport extent and directionality could be controlled by adjusting the electrode configuration. For instance, directional drug transport along the longitudinal axis was achieved in the butterfly-shaped tumor model. The maximum electric field magnitude across all configurations was 49.26 V/m, which was below the approximately 190 V/m axonal activation threshold reported for deep brain stimulation [37]. This threshold comparison serves as an approximate computational reference for the magnitude of the simulated electric field. Overall, these findings demonstrate the feasibility of iontophoretic drug delivery to brain tumors and suggest the need for patient-specific computational simulation to identify effective electrode configurations.

Each of the three configuration types exhibited distinct characteristics in drug transport. The scalp electrode configurations offered the advantage of non-invasiveness and thus allowed the use of a higher injection current based on established safety guidelines for tES [34]. However, the electric field within the tumor was substantially attenuated as the current passed through multiple tissue layers between the scalp and the tumor (Figure 3a,c). In particular, the shunting effect of the highly conductive CSF layer caused a large portion of the current to flow preferentially through the CSF rather than reaching the tumor [24]. As a result, the scalp electrode configurations yielded the lowest TC among the three configuration types in both tumor models. In addition, the scalp electrode configurations produced directional electric fields within the tumor, as the current must flow between the anodes and cathodes positioned on the scalp. This inherent directionality limited the ability to achieve uniform radial drug spreading, which was especially disadvantageous for compact tumor morphologies (e.g., Figure 4).

**Figure 6:**
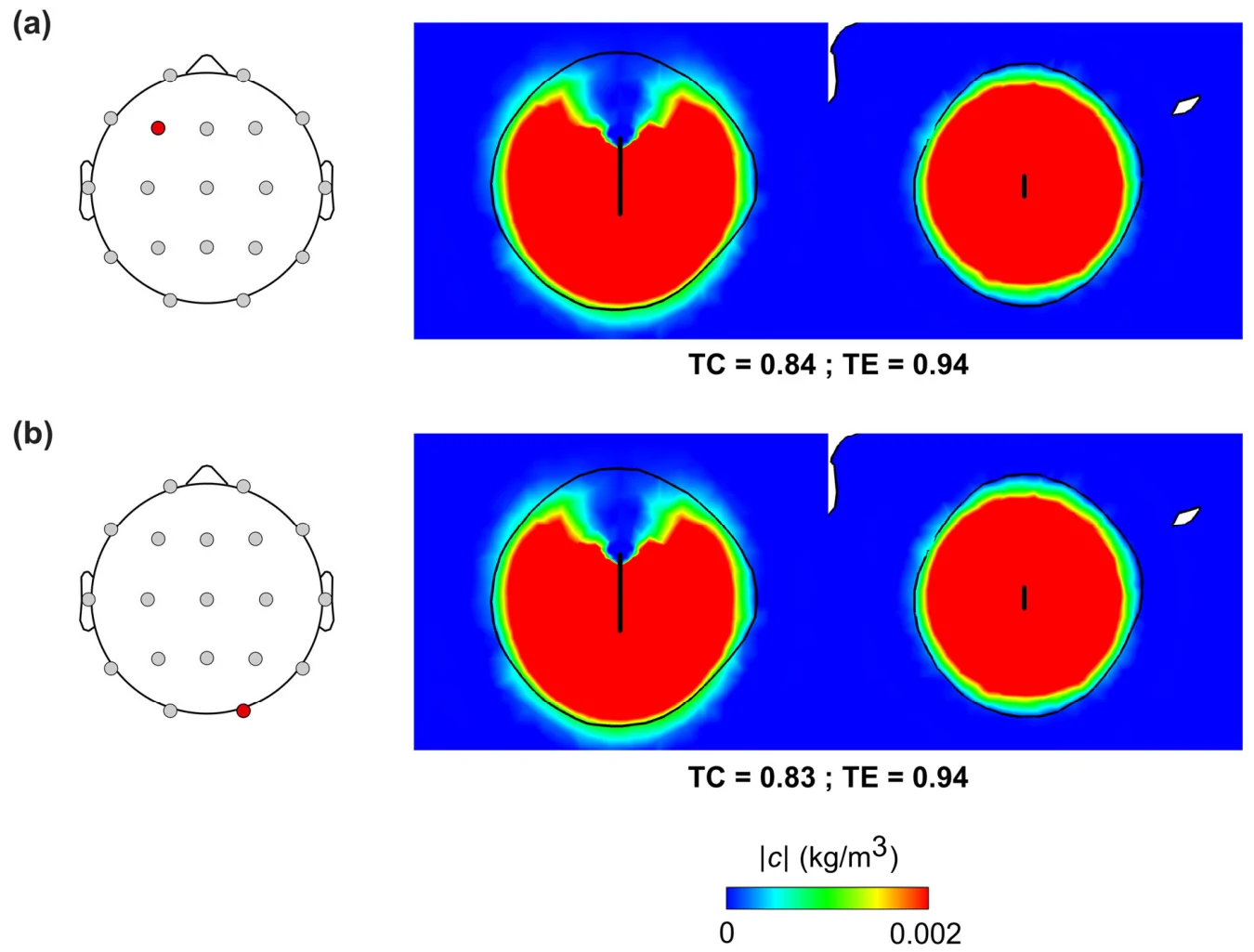
Effect of scalp anode position on drug distribution in the combined catheter and scalp electrode configuration for the spherical tumor model. The scalp anode position is indicated by the red marker on the 10–20 system electrode layout (left). Drug concentration distributions at 180 min are shown in coronal (middle) and transverse (right) cross-sections. In both cases, the catheter cathode was placed at the tumor center. The tumor coverage (TC) and targeting efficiency (TE) values are indicated below each panel. (**a**) Result with the scalp anode nearest to the tumor (F3). (**b**) Result with the scalp anode most distant from the tumor (O2).

The combined catheter and scalp electrode configurations produced more focused electric field distributions than the scalp electrode configurations. High electric field intensities were confined primarily around the catheter electrode and beneath the scalp electrode (Figure 3b,d). In both tumor models, the electric field around the catheter was radially distributed, and the drug also spread radially from the injection region (Figure 4 and Figure 5). To evaluate whether this radial pattern was independent of the scalp anode position, an additional simulation was performed in the spherical tumor model. With the catheter electrode fixed at the tumor center, the scalp anode was changed from the nearest electrode to the tumor (F3) to the most distant one (O2). As shown in Figure 6, the drug distributions were nearly identical, with a TC difference of only 0.01. This finding confirms that radial drug transport is consistently observed in this configuration type regardless of the scalp anode position. The high conductivity of the CSF layer may explain this observation. The current from the scalp anode spreads along the CSF rather than directly reaching the catheter in a specific direction. In the spherical tumor model, this radial spreading effectively delivered the drug throughout the tumor volume. In the butterfly-shaped tumor model, however, the same radial spreading caused substantial drug loss beyond the tumor boundary. These results suggest that the effectiveness of the combined catheter and scalp electrode configuration depends on the tumor morphology.

The catheter electrode configurations produced the most spatially confined electric fields among the three configuration types (Figure 3e,f). Because all electrodes were positioned within the tumor, the electric field between them enabled directional control of drug transport. In the two-catheter configuration, directional drug transport toward the anode was observed. However, the coverage remained limited due to the long transport distance across the entire length of the tumor. The three-catheter configuration addressed this limitation by placing the injection region at the center of the tumor. The bilateral electric field generated by the central cathode and two lateral anodes delivered the drug in both directions along the longitudinal axis. This configuration achieved the highest TC for the butterfly-shaped tumor model while maintaining a higher TE than the combined configuration. However, even with the three-catheter configuration, TC reached only 0.49, indicating that approximately half of the modeled tumor remained below the selected concentration threshold. This limited coverage highlights the need for further optimization of electrode placement and drug administration strategies for irregular tumor geometries. These results suggest that catheter electrode configurations may be advantageous for elongated tumors, although further optimization is needed to improve tumor coverage.

Previous studies of local brain drug delivery have primarily focused on convection-enhanced delivery, in which drug distribution is governed by pressure-driven convection and diffusion [9,10,11,12]. Our previous study demonstrated that iontophoresis can provide directional transport of gadobutrol in rat brain tissue [13], but did not address human brain tumors or the influence of tumor morphology on electrode configuration. Computational studies of glioblastoma have also shown that tumor anatomy and electrode placement can substantially affect intracranial electric field distributions [21,25]. However, these studies focused on electric field exposure rather than electrically guided drug transport. The present study extends these previous approaches by coupling electric field modeling with spatiotemporal drug transport and systematically comparing multiple electrode configurations across two distinct tumor morphologies.

This study has several limitations that should be addressed in future research. First, the present study was based on a single anatomical head model and two idealized tumor geometries. This limits the generalizability of the findings. Interindividual differences in skull thickness, CSF volume, brain anatomy, and tumor location may alter current pathways and electric-field distributions [21,24]. These differences may consequently affect the predicted drug distribution. The spherical and butterfly-shaped tumors were designed to represent compact unilateral and bilaterally extending tumors, respectively. However, these models do not capture the full heterogeneity of glioblastoma. Patient tumors can differ substantially in size, infiltration pattern, necrotic structure, and location [38]. In addition, the tumor shell was modeled using spatially uniform properties and did not account for patient-specific variations in cellular density, extracellular matrix composition, or interstitial transport properties. Such intratumoral heterogeneity may influence drug transport and alter the predicted drug distribution. Therefore, the electrode configurations identified in the present models should not be directly generalized to all patients. Future studies should evaluate the proposed framework using multiple patient-specific head models with MRI-derived tumor geometries. Electrode configurations should also be re-optimized for each individual anatomy and tumor morphology. Second, all tissues were assumed to be homogeneous and isotropic. However, white matter exhibits anisotropic electrical conductivity [39] and diffusion properties [40] along its fiber tracts. The influence of this simplification is expected to be limited for catheter-only configurations because the strongest electric fields are concentrated within the tumor. In the combined catheter–scalp configuration, Figure 6 also showed nearly identical drug distributions despite a large change in scalp anode position, suggesting that drug transport was mainly governed by the local electric field around the catheter. Nevertheless, white matter anisotropy was not explicitly modeled and may affect current pathways, particularly in configurations relying solely on scalp electrodes. Future work should therefore incorporate anisotropic tissue properties to directly evaluate their effects on electrode optimization and tumor coverage.

Additional limitations are related to the drug transport model. Several transport parameters were not directly measured in human brain tissue. The diffusion coefficients and charge number of gadobutrol were estimated from the available literature, while the empirical correction factors (α and γ) were adopted from our previous rat-brain study [13]. To assess the robustness of the results to these parameter uncertainties, sensitivity analyses were performed by varying *α* and γ from 50% to 150% of their nominal values. Although the absolute TC and TE values changed, the main configuration-dependent trends were preserved across the tested conditions (Figure S1). Nevertheless, direct measurement and calibration of these parameters in human brain tissue would improve the quantitative reliability of the model. Another limitation is that gadobutrol was used as a model compound rather than an actual therapeutic agent for glioblastoma. Physicochemical and transport properties, including charge, molecular size, diffusion coefficient, tissue binding, and therapeutically relevant concentration, can differ substantially among compounds. Therefore, the electrode configurations identified in the present study may not directly apply to anticancer agents, and future studies should evaluate therapeutic compounds using drug-specific transport parameters. In addition, maintaining a constant drug concentration at the injection region represents sustained drug supply and does not account for depletion of a finite dose. A condition with a finite dose or a concentration that decreases over time could reduce TC at later time points and may also alter TE. Incorporating time-dependent injection conditions would better reflect realistic delivery scenarios.

## 5. Conclusions

We demonstrated the feasibility of iontophoretic drug delivery to human brain tumors through computational simulations. Unlike passive diffusion and CED, this approach enabled directional control of drug transport along the applied electric field. The results also revealed that the effective electrode configuration differed depending on tumor morphology, highlighting the potential value of patient-specific simulation-based optimization for treatment planning. This work provides a computational foundation for future patient-specific modeling and experimental studies of iontophoretic drug delivery to human brain tumors.

## Figures and Tables

**Table 1:** Conductivity and diffusion coefficient of brain tissues and electrodes.

Region	Conductivity, *σ* (S/m)	Diffusion Coefficient, *D* (×10^−10^ m^2^/s)
Scalp	0.25	0
Skull	0.015	0
CSF	1.79	8.8
Gray matter	0.276	2.2
White matter	0.126	2.2
Tumor shell	0.24	2.2
Necrotic core	1.0	4.4
Electrode	5.21	0

## Data Availability

Please contact the corresponding author (ich@hanyang.ac.kr) for data requests.

## Supplementary Materials

The following supporting information can be downloaded at: https://www.mdpi.com/article/10.3390/pharmaceutics18101300/s1, Figure S1: Sensitivity of tumor coverage (TC) and targeting efficiency (TE) to variations in the time factor (*α*) and charge factor (*γ*); Figure S2: Sensitivity of tumor coverage (TC) and targeting efficiency (TE) to variations in tumor conductivity; Table S1: Sensitivity analysis of tumor coverage (TC) across different concentration thresholds; Table S2: Sensitivity of tumor coverage (TC) and targeting efficiency (TE) to variations in the time factor (*α*) and charge factor (*γ*); Table S3: Sensitivity of tumor coverage (TC) and targeting efficiency (TE) to variations in tumor conductivity.
