# Supplementary material for: In Silico Investigation of Electrically Guided Drug Transport Toward Morphology-Specific Brain Tumor Treatment
Source: Pharmaceutics. 2026 Oct 14;18(10):1300. doi: 10.3390/pharmaceutics18101300 (PMC13708410; doi:10.3390/pharmaceutics18101300)
Supplement: Supplementary file 1 [file pharmaceutics-18-01300-s001.zip › pharmaceutics-4573824-supplementary.pdf]

Table S1. Sensitivity analysis of tumor coverage (TC) across different concentration thresholds. TC was calculated using threshold concentrations of 5%, 10%, and 20% of the administered concentration (0.01 kg/m³). Bold values indicate the highest TC for each tumor model at each threshold.

Tumor type	Configuration	TC_5%	TC_10%	TC_20%
Spherical tumor	Passive diffusion	0.18	0.13	0.08
	Scalp electrodes	0.24	0.18	0.11
	1 catheter electrode<br>1 scalp electrode	**0.93**	**0.84**	**0.64**
Butterfly-shaped tumor	Scalp electrodes	0.17	0.13	0.09
	1 catheter electrode<br>2 scalp electrodes	0.50	0.45	0.38
	2 catheter electrodes	0.35	0.29	0.22
	3 catheter electrodes	**0.55**	**0.49**	**0.41**

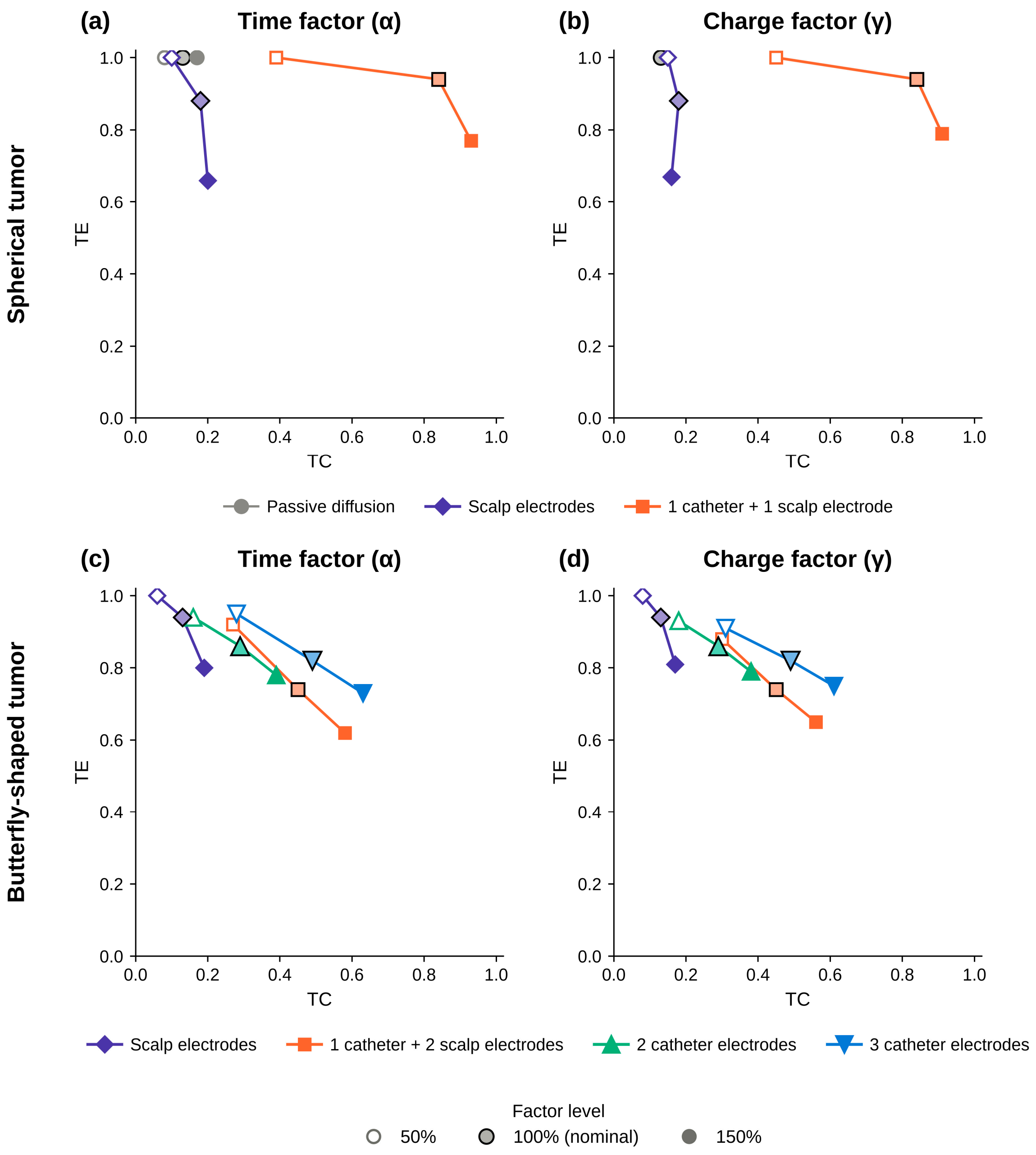

Figure S1. Sensitivity of tumor coverage (TC) and targeting efficiency (TE) to variations in the time factor (α) and charge factor (γ). Each factor was varied independently to 50%, 100%, and 150% of their nominal values ($\alpha = 0.5$ and $\gamma = 25$), while the other factor was held at its nominal value. (a, b) Spherical tumor model and (c, d) butterfly-shaped tumor model. (a, c) Time-factor sensitivity and (b, d) charge-factor sensitivity. Electrode configurations and applied current distributions were fixed across all conditions. Passive diffusion varied with the time factor because α affects the overall transport timescale, whereas it was unaffected by the charge factor because γ acts only on the electromigration term.

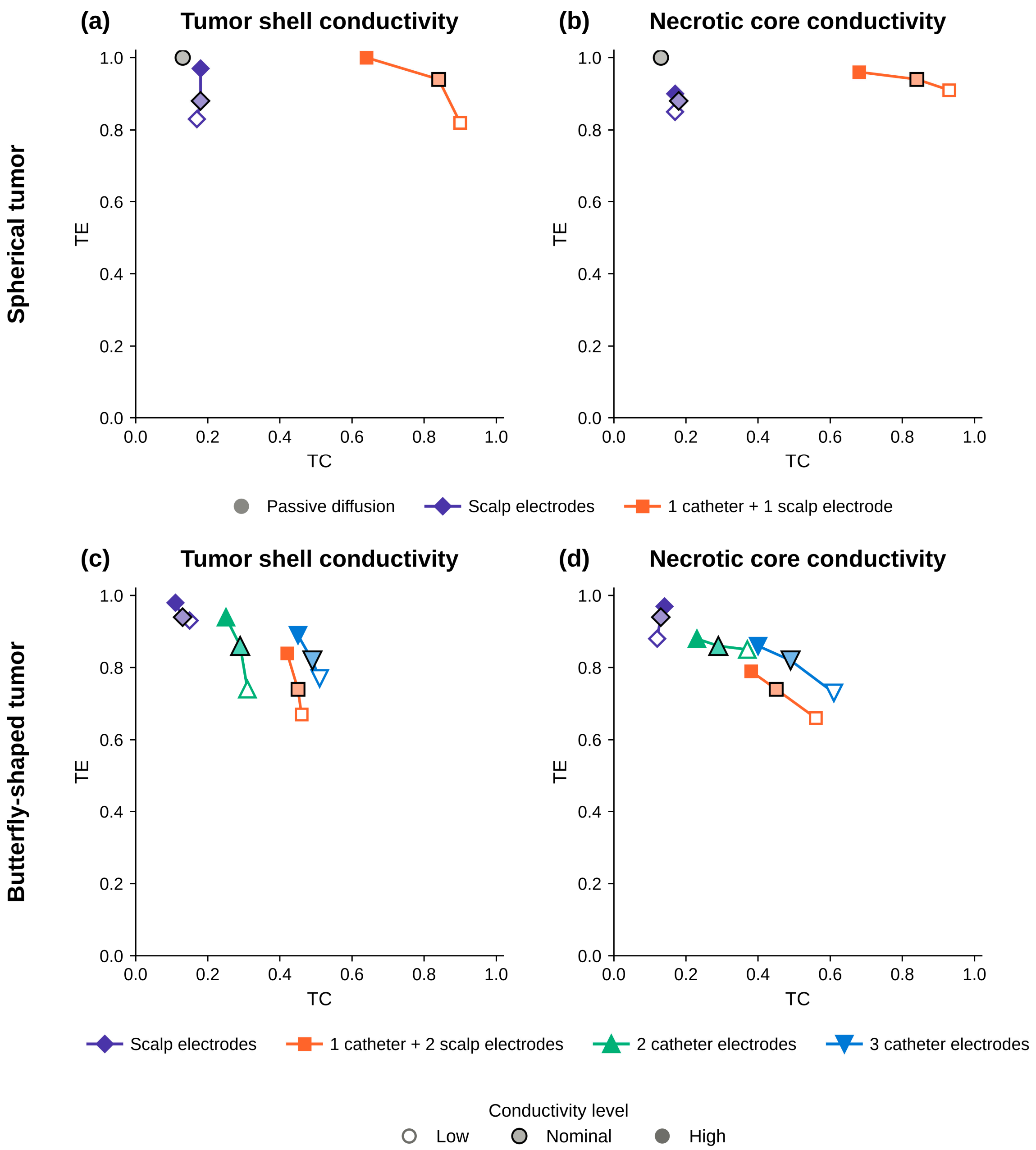

Figure S2. Sensitivity of tumor coverage (TC) and targeting efficiency (TE) to variations in tumor conductivity. The conductivities of the tumor shell and necrotic core were varied independently while the other conductivity values were held at their nominal values. The tumor shell conductivity was varied among 0.15, 0.24, and 0.50 S/m, corresponding to the low, nominal, and high conditions, respectively. The necrotic core conductivity was varied among 0.50, 1.00, and 1.50 S/m. (a, b) Spherical tumor model and (c, d) butterfly-shaped tumor model. (a, c) Sensitivity to tumor shell conductivity and (b, d) sensitivity to necrotic core conductivity. Electrode locations and applied current distributions were fixed to those determined under the nominal conductivity condition. Connected markers indicate changes in TC and TE across the three conductivity levels. Passive diffusion is represented by a single point because it is unaffected by electrical conductivity in the absence of an applied electric field.

Table S2. Sensitivity of tumor coverage (TC) and targeting efficiency (TE) to variations in the time factor (α) and charge factor (γ). Each factor was varied independently to 50% and 150% of its nominal value ($\alpha = 0.5$ and $\gamma = 25$), while the other factor was held at its nominal value. Values were evaluated at the final simulation time of 180 min for the spherical tumor model and 300 min for the butterfly-shaped tumor model.

Metric	Tumor type	Configuration	Nominal	α = 0.25 (50%)	α = 0.75 (150%)	γ = 12.5 (50%)	γ = 37.5 (150%)
TC	Spherical tumor	Passive diffusion	0.13	0.08	0.17	0.13	0.13
		Scalp electrodes	0.18	0.10	0.20	0.15	0.16
		1 catheter electrode<br>1 scalp electrode	0.84	0.39	0.93	0.45	0.91
	Butterfly-shaped tumor	Scalp electrodes	0.13	0.06	0.19	0.08	0.17
		1 catheter electrode<br>2 scalp electrodes	0.45	0.27	0.58	0.30	0.56
		2 catheter electrodes	0.29	0.16	0.39	0.18	0.38
		3 catheter electrodes	0.49	0.28	0.63	0.31	0.61
TE	Spherical tumor	Passive diffusion	1.00	1.00	1.00	1.00	1.00
		Scalp electrodes	0.88	1.00	0.66	1.00	0.67
		1 catheter electrode<br>1 scalp electrode	0.94	1.00	0.77	1.00	0.79
	Butterfly-shaped tumor	Scalp electrodes	0.94	1.00	0.80	1.00	0.81
		1 catheter electrode<br>2 scalp electrodes	0.74	0.92	0.62	0.88	0.65
		2 catheter electrodes	0.86	0.94	0.78	0.93	0.79
		3 catheter electrodes	0.82	0.95	0.73	0.91	0.75

Table S3. Sensitivity of tumor coverage (TC) and targeting efficiency (TE) to variations in tumor conductivity. The conductivity of the tumor shell and necrotic core was varied independently while all other conductivity values were held at their nominal values. The nominal conductivities were 0.24 S/m for the tumor shell and 1.00 S/m for the necrotic core. Electrode configurations and applied current distributions were fixed to those determined under the nominal conductivity condition. Values were evaluated at the final simulation time of 180 min for the spherical tumor model and 300 min for the butterfly-shaped tumor model.

Metric	Tumor type	Configuration	Nominal	Tumor shell 0.15 S/m	Tumor shell 0.50 S/m	Necrotic core 0.50 S/m	Necrotic core 1.50 S/m
TC	Spherical tumor	Passive diffusion	0.13	0.13	0.13	0.13	0.13
		Scalp electrodes	0.18	0.17	0.18	0.17	0.17
		1 catheter electrode 1 scalp electrode	0.84	0.90	0.64	0.93	0.68
	Butterfly-shaped tumor	Scalp electrodes	0.13	0.15	0.11	0.12	0.14
		1 catheter electrode 2 scalp electrodes	0.45	0.46	0.42	0.56	0.38
		2 catheter electrodes	0.29	0.31	0.25	0.37	0.23
		3 catheter electrodes	0.49	0.51	0.45	0.61	0.40
TE	Spherical tumor	Passive diffusion	1.00	1.00	1.00	1.00	1.00
		Scalp electrodes	0.88	0.83	0.97	0.85	0.90
		1 catheter electrode 1 scalp electrode	0.94	0.82	1.00	0.91	0.96
	Butterfly-shaped tumor	Scalp electrodes	0.94	0.93	0.98	0.88	0.97
		1 catheter electrode 2 scalp electrodes	0.74	0.67	0.84	0.66	0.79
		2 catheter electrodes	0.86	0.74	0.94	0.85	0.88
		3 catheter electrodes	0.82	0.77	0.89	0.73	0.86
